# A novel cell exclusion zone assay with a barrier made from room temperature vulcanizing silicone rubber

**DOI:** 10.1371/journal.pone.0190198

**Published:** 2017-12-21

**Authors:** Yusuke Shiode, Yuki Morizane, Ryo Matoba, Masayuki Hirano, Shinichiro Doi, Shinji Toshima, Ryoichi Araki, Mika Hosogi, Kosuke Takahashi, Yuki Kanzaki, Tomoko Yonezawa, Fumio Shiraga

**Affiliations:** 1 Department of Ophthalmology, Okayama University Graduate School of Medicine, Dentistry and Pharmaceutical Sciences, Okayama, Japan; 2 Department of Molecular Biology and Biochemistry, Okayama University Graduate School of Medicine, Dentistry and Pharmaceutical Sciences, Okayama, Japan; Massachusetts Eye & Ear Infirmary, Harvard Medical School, UNITED STATES

## Abstract

**Objective:**

To examine the usefulness of room temperature vulcanizing (RTV) silicone rubber as a barrier material for cell exclusion zone assays.

**Methods:**

We created barriers using three types of RTV silicone rubber with differing viscosities. We then assessed the adherence of these barriers to culture dishes and their ease of removal from the dishes. We tested the effect of the newly created barriers on the extracellular matrix (ECM) protein fibronectin by attaching and then removing them from fibronectin-coated culture dishes. We also conducted cell exclusion zone assays with MIO-M1 cells using this new barrier in order to measure cell migration. We used real time reverse transcription polymerase chain reaction (RT-PCR) and immunohistochemical staining to measure the effect of fibronectin on MIO-M1 cell migration and the effect of migration (with fibronectin coating) on *basic fibroblast growth factor* (*bFGF*) expression in MIO-M1 cells.

**Results:**

Of the three types of RTV silicon rubber tested, KE-3495-T was the best in terms of adherence to the dish and ease of removal from the dish. When barrier attachment and removal tests were performed, this rubber type did not have an effect on the fibronectin that coated the dish. In the cell exclusion assay, removal of the barrier revealed that a cell-free area with a distinct margin had been created, which allowed us to conduct a quantitative assessment of migration. Fibronectin significantly promoted the migration of MIO-M1 cells (*P* = 0.02). In addition, both real time RT-PCR and immunohistological staining indicated that *bFGF* expression in migrating MIO-M1 cells was significantly higher than that in non-migrating cells (*P* = 0.03).

**Conclusions:**

RTV silicone rubber can be used to create an effective barrier in cell exclusion zone assays and allows simple and low-cost multi-parametric analysis of cell migration.

## Introduction

Cell migration plays a pivotal role in both physiological and pathological processes such as embryonic development, wound healing, cancer metastasis, and angiogenesis [1–3]. One of the important factors determining cell motility is the extracellular matrix (ECM). The ECM is composed of glycoproteins, including collagens, fibronectins, laminins, and proteoglycans. ECM proteins provide a structural basis that supports cell migration. ECM proteins also function as a reservoir for growth factors and bind to the intracellular cytoskeleton by integrin adhesion receptors, thereby affecting cell adhesion, polarity, and migration [4–7]. Therefore, elucidating the effects of various ECM proteins on cell motility may aid in the development of novel therapies and drugs.

A variety of *in vitro* cell migration assays have previously been devised as methods of investigating cell motility [8–10]. However, the cell exclusion zone assay is currently the only method that allows investigation of the effects of ECM proteins on cell motility [11]. Similar to scratch assays and microfluidic assays, the cell exclusion zone assay is a 2D cell migration method characterized by the use of a barrier to prevent the engraftment of cultured cells. After coating a culture dish with ECM protein, the barrier is put into position, and the cells are seeded. Once the cells are confluent, the barrier is removed and the resulting cell motility can be observed. However, the cell exclusion zone assay kits that are currently commercially available consist of either a 96-well plate or a 384-well plate. Because the dimensions of the assay are therefore small, the only variable that can be investigated is cell motility area [12]. In addition, because the plates are sold with specific ECM protein coatings already applied, the choice of ECM proteins that can be studied is also limited. Finally, commercially available kits are also costly.

Therefore, a simple method to create barriers for use in cell motility arrays would be useful for a wide range of research. This barrier would require two characteristics: a high degree of adhesiveness to the culture dish as well as the ability to be easily removed from the same dish. In order to prevent cell migration, the base of the barrier must adhere to the dish without any gaps. However, it is also necessary to be able to remove the barrier completely from the culture dish to prevent remnants of the barrier from interfering with cell migration once it has begun. In this study, we developed a method to create a barrier of room temperature vulcanizing (RTV) silicone rubber for use in cell exclusion zone assays. We used the resulting barriers to investigate the effect of fibronectin on MIO-M1 cell migration as well as the influence of MIO-M1 cell migration on *basic fibroblast growth factor* (*bFGF*) expression.

## Materials and methods

### Chemicals and antibodies

RTV silicone rubber compounds (KE-3423, KE-3495-T, and KE-3497-T) were purchased from Shin-Etsu Chemical Co., Ltd. (Tokyo, Japan). Anti-fibronectin antibody (No. ab2413) and anti-basic fibroblast growth factor (FGF) primary antibody (No. ab8880) were purchased from Abcam (Cambridge, UK). The secondary antibody, Alexa Fluor 488 goat anti-rabbit IgG (No. A-11034), was purchased from Thermo Fisher Scientific (Waltham, MA, USA). Horseradish peroxidase (HRP)-conjugated anti-rabbit IgG (No. ab6721) was purchased from Abcam. Recombinant fibronectin (No. 354008) was purchased from BD Biosciences (Franklin Lakes, NJ, USA). Dulbecco’s modified eagle’s medium (DMEM), fetal bovine serum (FBS), and penicillin/streptomycin were purchased from Thermo Fisher Scientific. Endothelial Cell Growth Medium was purchased from PromoCell (Heidelberg, Germany).

### Cell culture

MIO-M1 cells, which are immortalized Müller glial cells, were generously donated by Dr. Astrid Limb (University College London, London, UK), and human umbilical vein endothelial cells (HUVEC) were purchased from PromoCell. These cells were cultured in appropriate media (MIO-M1 cells; DMEM and 10% FBS supplemented with 1% penicillin/streptomycin, HUVEC; Endothelial Cell Growth Medium) at 37°C in a humidified atmosphere of 5% CO_2_ and 95% air.

### Creation of the cell migration barrier using silicone rubber

**Fig 1:**
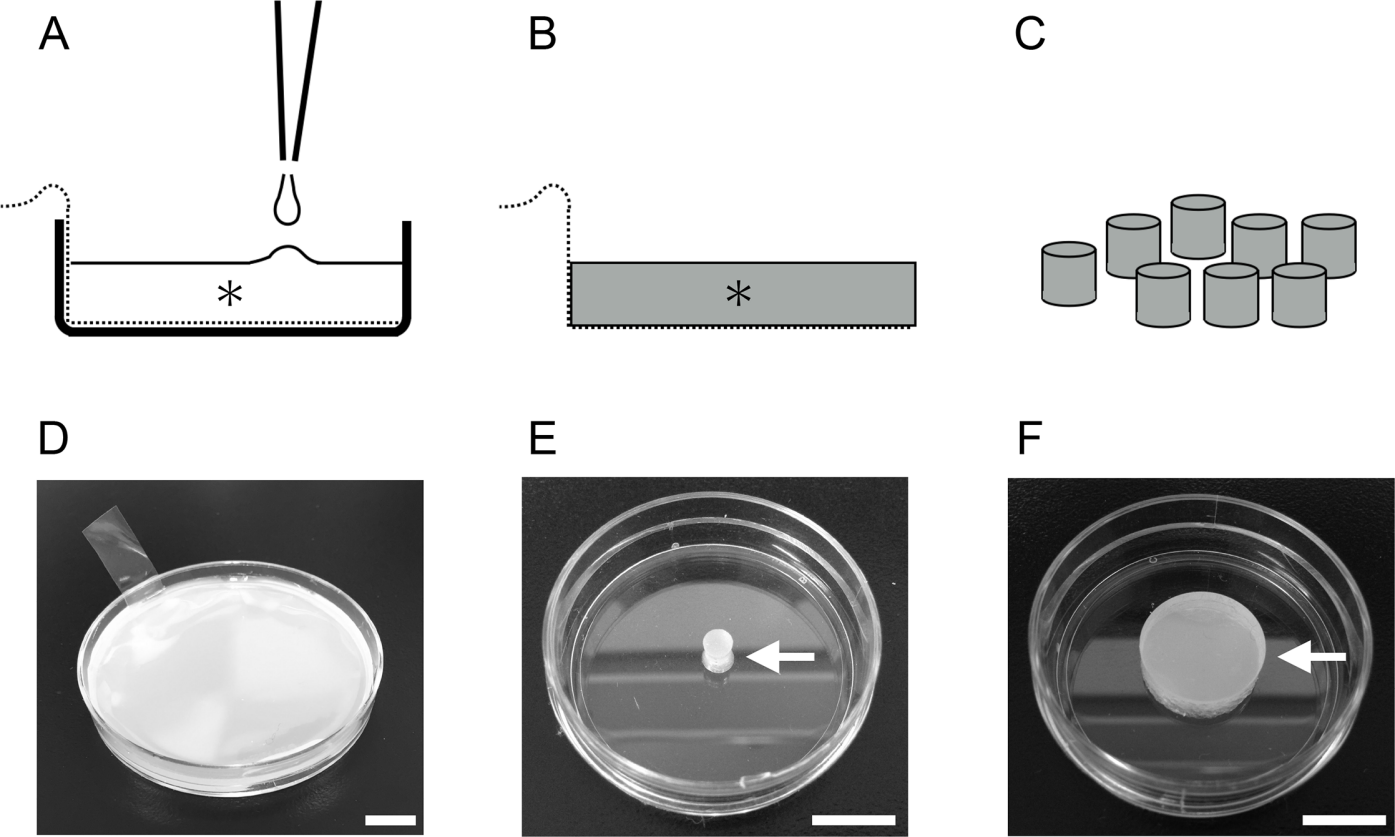
Creation of the cell migration barrier. We placed polypropylene film in the culture dish, poured 10 g of liquid RTV silicone rubber (asterisk) on top of this film, and let it harden overnight (A and D). We then removed the hardened RTV silicone rubber (asterisk) from the plate (B). After removing the polypropylene film, we used a skin trephine to carve out a cylindrical section for use as a barrier (C). Arrow E shows a 4-mm diameter barrier, and arrow F shows a 15-mm diameter barrier. Bars = 10 mm (S1 Video).

We placed a polypropylene film (Hybri-Bag, Cosmo Bio, Tokyo, Japan) on the bottom of a culture dish (60-mm diameter). We then poured 10 g of RTV silicone rubber on top of the film and left it to harden overnight in room air (Fig 1A and 1D). The hardened RTV silicone rubber and the polypropylene film were then pulled from the culture dish (Fig 1B). After removing the polypropylene film, we used a skin trephine (4-mm diameter; Dermapunch, Maruho, Osaka, Japan) or a leather punch (15-mm diameter; Daiso-sangyo, Higashihiroshima, Japan) to make a cylindrical barrier from the RTV silicone rubber (Fig 1C, 1E and 1F). The barrier was autoclaved for 20 minutes at 121°C before use in the cell migration assay (S1 Video).

### Cell migration assay with fibronectin coating

**Fig 2:**
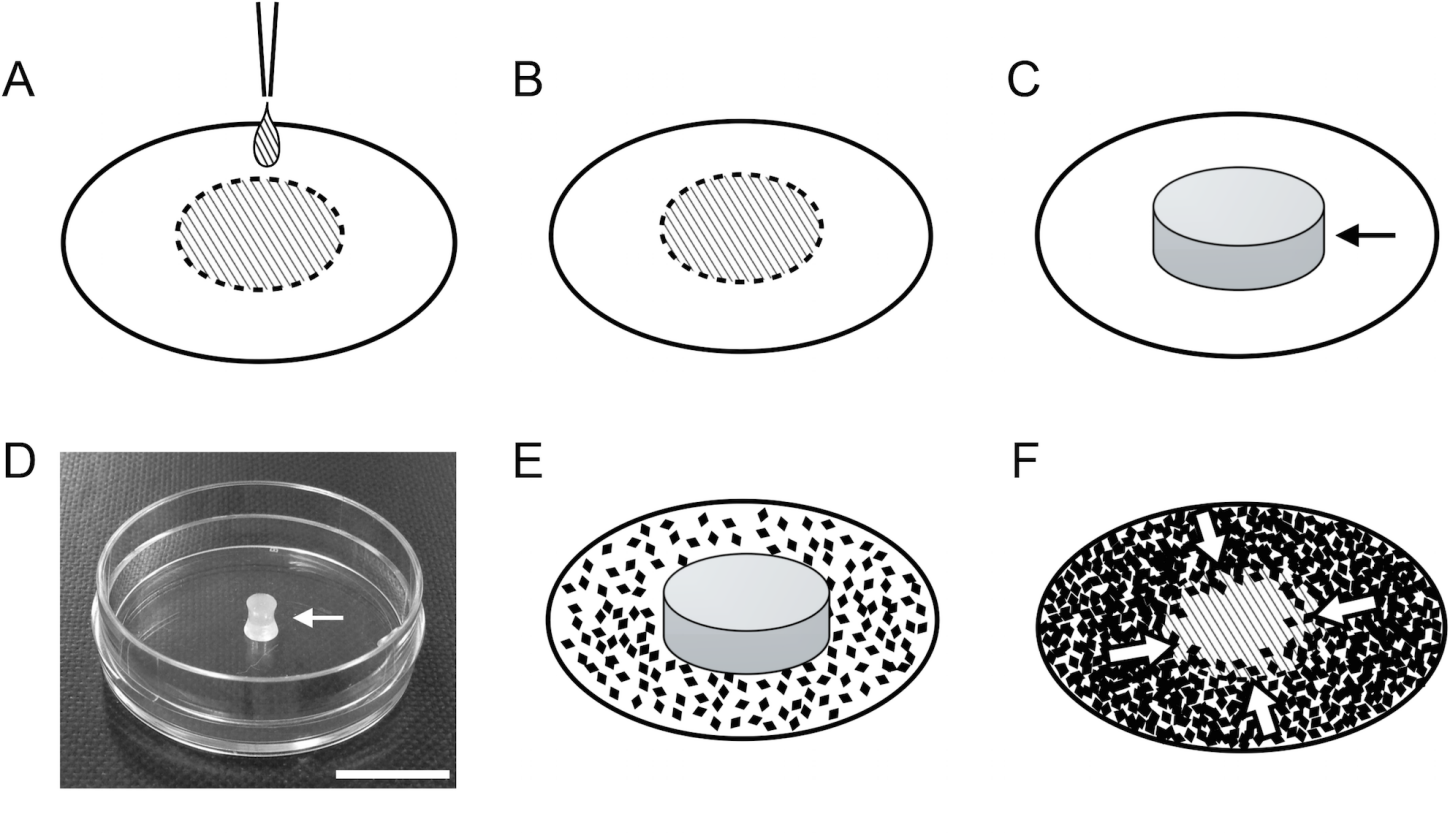
Cell migration assay technique designed to examine the effect of fibronectin on migration of the coating. Fibronectin was applied to the center of a culture dish so that its area was equal to the base area of the barrier (A). It was left to harden for 1 hour, and then the dish was washed to remove excess fibronectin using PBS (B). After the culture dish had dried, the barrier (arrow) was positioned so as to cover the coated area and then pressed in order to adhere it to the culture plate (C, D). Next, MIO-M1 cells were seeded on plates at 80% confluency (E). When the cells were 100% confluent, the barrier was removed to induce cell migration (F, arrows indicate direction of cell migration). Bar = 10 mm.

We placed fibronectin (50 μg/mL) into the center of a 35-mm diameter culture dish in order to create a 12.6 mm^2^ area, equal to the base area of the 4-mm diameter barrier (Fig 2A and 2B). Based on our observations, the amount of fibronectin required was approximately 10 μl. After letting this stand at room temperature for 1 hour, the dish was washed with phosphate buffered saline (PBS) to remove excess fibronectin. The fibronectin-coated area was then covered with the 4-mm diameter barrier, which was lightly pressed into the base of the culture dish to ensure proper adherence (Fig 2C and 2D). Next, the MIO-M1 cells were seeded on plates at 80% confluency (Fig 2E). When the cells reached 100% confluency, we carefully removed the barrier. Cells were then able to migrate toward the cell-free-area (Fig 2F). The cell-free area was photographed every 24 hours using a phase contrast microscope (CKX-41, Olympus, Japan). The area of the cell-free space was measured using Image J software (National Institutes of Health, Bethesda, MD) in order to calculate the area of cell migration.

### Reverse transcription polymerase chain reaction (RT- PCR)

Real time RT-PCR (StepOne, Thermo Fisher Scientific) was carried out according to the manufacturer’s protocol. A Taqman (Thermo Fisher Scientific) gene expression assay primer was used to quantify the mRNA of FGF2 (Hs00266645_m1).

### Fibronectin immunostaining of silicone barrier base and culture dish surface

The surface of the culture dish and the base of the silicone barrier were both fixed with 4% paraformaldehyde. Anti-fibronectin antibody (1:200) was left on both surfaces overnight at 4°C. The culture dish was then allowed to react with Alexa Fluor 488 goat anti-rabbit IgG in room air for 60 minutes, while the silicone barrier was allowed to react with HRP-conjugated anti-rabbit IgG under the same conditions. Images of the culture dish were acquired with a fluorescence microscope (FSX100, Olympus, Japan). Visualization of the silicone barrier was conducted using a DAB Universal Kit (Ventana Medical Systems), and observations were made using a stereoscopic microscope (OMS-800, Topcon, Tokyo, Japan).

### Immunostaining of MIO-M1 cells

Cells were cultured after attaching the barrier to a chamber slide (Nunc Lab-Tek Chamber Slide System 177429, Thermo Fisher Scientific, MA). We then removed the barrier and fixed the cells with 4% paraformaldehyde at least 48 hours after migration began. The cells were then processed with 0.25% TritonX-100, washed with PBS, and then left overnight at 4°C in order to react with anti-bFGF antibody (1:100). After this, the cells were washed once again with PBS and left for 30 minutes in room air to react with a secondary antibody (Alexa Fluor 488 goat anti-rabbit IgG). DAPI staining solution (Life Technologies, Grand Island, NY, USA) was used to stain cell nuclei and thus display the positions of the cells. After washing with PBS again, images were acquired using a fluorescence microscope.

### Statistical analysis

All experiments were repeated a minimum of three times. All data are expressed as mean ± standard error (SE). Statistical differences between two groups were analyzed using the unpaired Student’s t test. Multiple group comparisons were performed by one-way analysis of variance with Bonferroni’s test. Differences were considered significant at p < 0.05.

## Results

### Investigation of the RTV silicone rubber that was suitable as a barrier used in cell migration assays

In order to discover a material for use as a barrier in cell exclusion zone assays, we investigated 3 types of RTV silicone rubber with differing viscosities (KE-3497-T, KE-3423 and KE-3495-T). The characteristics of these types of silicone rubber are shown in Table 1. The base of the barrier had to be smooth to ensure that it was able to adhere to the dish without any gaps. Therefore, it was necessary that the silicone rubber harden evenly within its container (in this study, a 60 mm culture dish was used for this purpose). As shown in Table 1, the viscosity of KE-3497-T when in the liquid state was higher than that of either KE-3423 or KE-3495-T. As a result, KE-3497-T did not spread evenly within its container. However, KE-3423 and KE-3495-T hardened uniformly in the 60 mm culture dish. Therefore, 4-mm diameter barriers were created using KE-3423 and KE-3495-T. These were then attached to culture dishes and then removed from the dishes after 24 hours. We were able to easily and completely remove KE-3495-T from the dish, but KE-3423 was so tenaciously adhered that pieces of rubber remained on the dish surface after its removal. Based on the above results, we concluded that KE-3495-T was the most suitable RTV silicone rubber type for use as a barrier for cell exclusion zone assays. All subsequent experiments were performed using barriers made with KE-3495-T.

**Table 1:** Characteristics of room temperature vulcanizing silicone rubber types used in this study. The data are based on the product catalog from Shin-Etsu silicone.

		KE-3423	KE-3495-T	KE-3497-T
Liquid State	Viscosity (Pa·s)	0.6	5.5	40
	Hardness (Durometer; A)	20	30	35
	Density 23°C (g/cm^3^)	0.98	1.03	1.07
Solid State	Hardened uniformly	✓	✓	X
	Easily removable from cell culture dish	X	✓	✓

### The effect of attaching and removing the silicone barrier on the culture dish fibronectin coating

**Fig 3:**
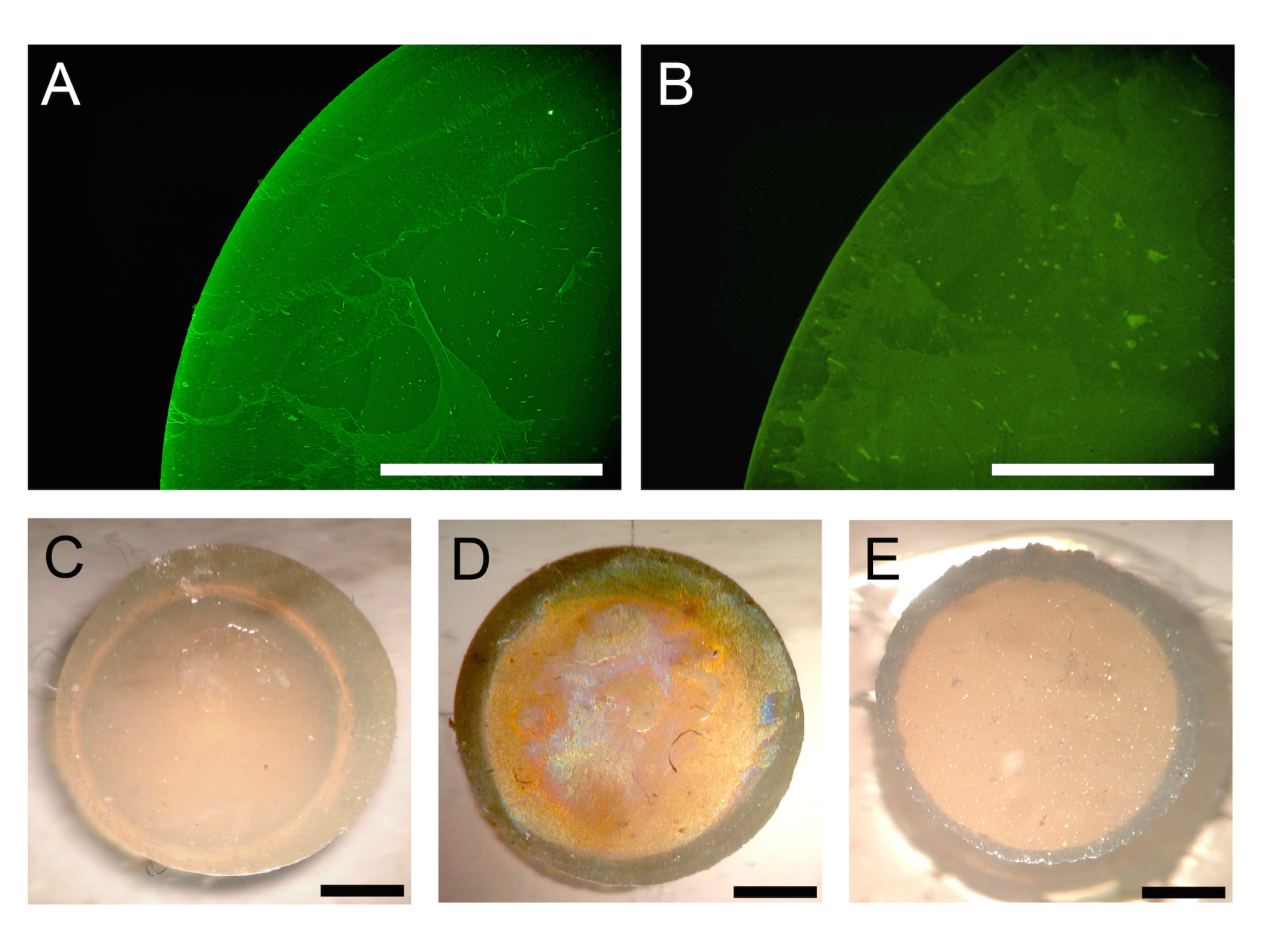
The effect of attaching and removing the silicone barrier on fibronectin coating. A: Immunostaining of a culture dish that was coated with fibronectin. The fibronectin is indicated by green fluorescent light. B: Image of fibronectin after immunostaining a culture dish. The barrier of the culture dish was adhered after coating the dish with fibronectin and then removed after 24 hours. The fibronectin coating remained intact as in A. C-E: Images of fibronectin immunostained barrier bases (DAB method). C: A barrier base with no coating (negative control). D: A barrier base with a fibronectin coating (positive control). E: The base of a barrier that had been removed 24 hours after being attached to a fibronectin-coated culture dish. No fibronectin adhered to the barrier base. Bars = 1 mm.

The cell exclusion zone assay is used to investigate how the extracellular matrix affects cell migration. This assay is performed by coating culture dishes with extracellular matrix proteins, attaching barriers in order to impede cell migration, and then inducing cell migration by removing the barriers. Therefore, the barrier must be attached and removed without damaging the extracellular matrix that is used as a coating. Thus, we used immunostaining to assess any changes in the extracellular matrix coating after attaching and removing KE-3495-T barriers. We coated the surface of a culture dish with fibronectin, attached the barrier, and then removed the barrier after 24 hours. We then stained the culture dish surface with fibronectin antibodies and observed that the fibronectin coating remained intact (Fig 3A and 3B). In addition, we stained the base of the removed barrier with fibronectin antibodies and observed that there was no attached fibronectin (Fig 3E). The above results indicate that attachment and removal of the barrier does not affect the coating on the culture dish.

### The effect of fibronectin on MIO-M1 cell migration

**Fig 4:**
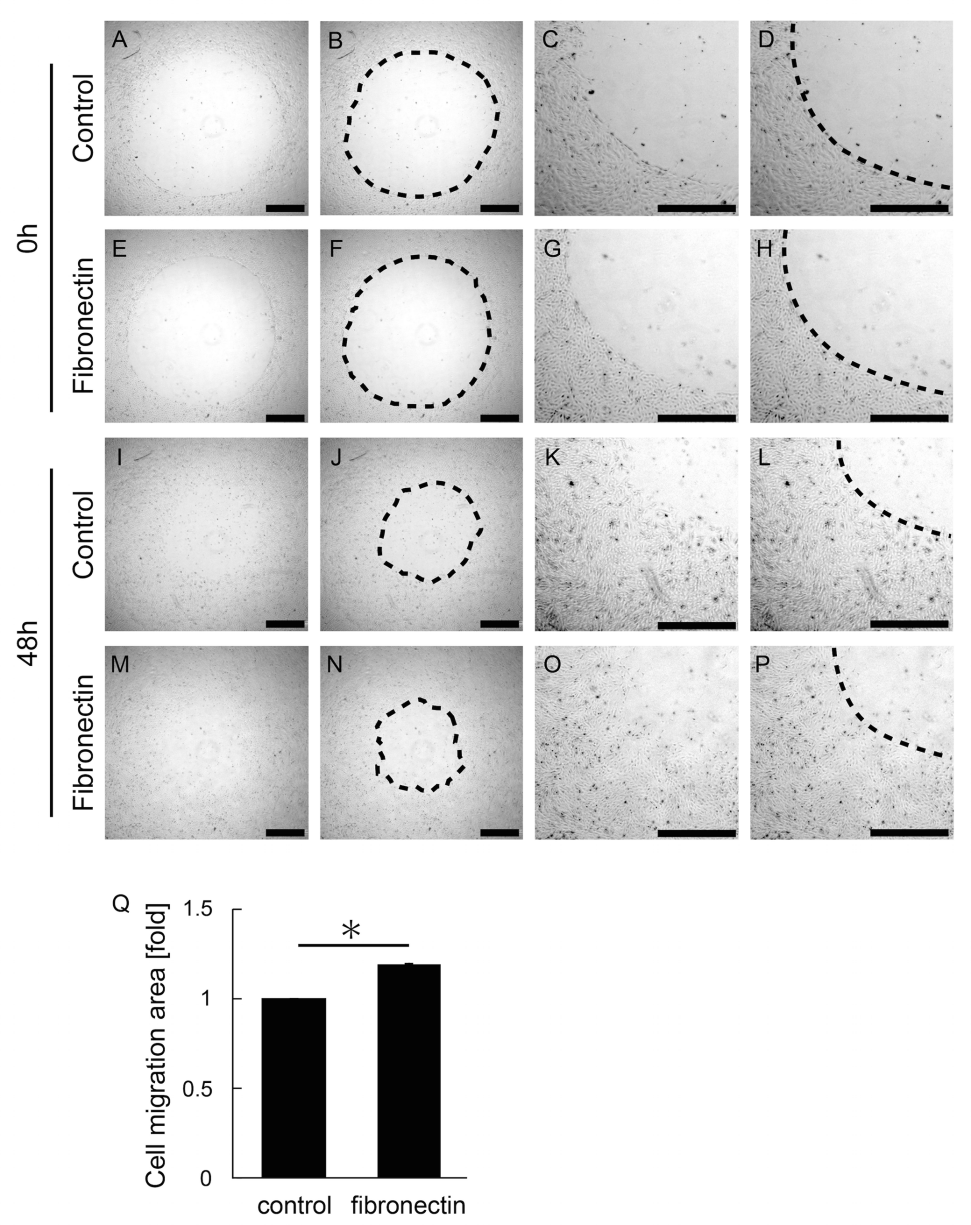
The effect of fibronectin on cell migration. A-P: Phase contrast microscopic images of MIO-M1 cells that have undergone a migrating assay. A-H show an image that was photographed immediately after removing the barrier, while I-P show an image taken 48 hours after removing the barrier. A-D and I-L show uncoated culture dishes, while E-H and M-P show culture dishes coated with fibronectin. C, G, K, and O each show an enlarged section of A, E, I, and M, respectively. Each dotted line in B, D, F, H, J, L, N, and P indicates the margin of the cell-free area in the image to the left of each of these panels. Bars = 1 mm. Q shows the migration area on a control dish and a fibronectin-coated dish at 48 hours after barrier removal. The migration area on the fibronectin-coated dish was significantly larger than that on the control dish. *P<0.05.

In order to investigate the effect of fibronectin on MIO-M1 cell migration and to validate the use of our RTV silicone rubber barrier, we coated the surface of a culture dish with fibronectin and attached the barrier. We then seeded the dish with MIO-M1 cells. Once they became confluent, we removed the barrier (Fig 4A–4H) and assessed the area of migration after 48 hours (Fig 4I–4P). As shown in Fig 4A–4H, cell migration was prevented by the barrier in both the uncoated control culture dish (Fig 4A–4D) and the fibronectin-coated dish (Fig 4E–4H). That is, there was a clear border between the cells and the area within which migration was prevented by the barrier. This indicates that the barrier completely prevented cell migration. The ratio of cell migration area in the fibronectin-coated dish compared with the control dish was 1.19±0.01 (*P* = 0.02, Fig 4Q). Therefore, we confirmed that in comparison to control conditions (i.e., an uncoated culture dish), the fibronectin led to significant promotion of MIO-M1 cell migration.

### The effect of cell migration on bFGF production in MIO-M1 cells

**Fig 5:**
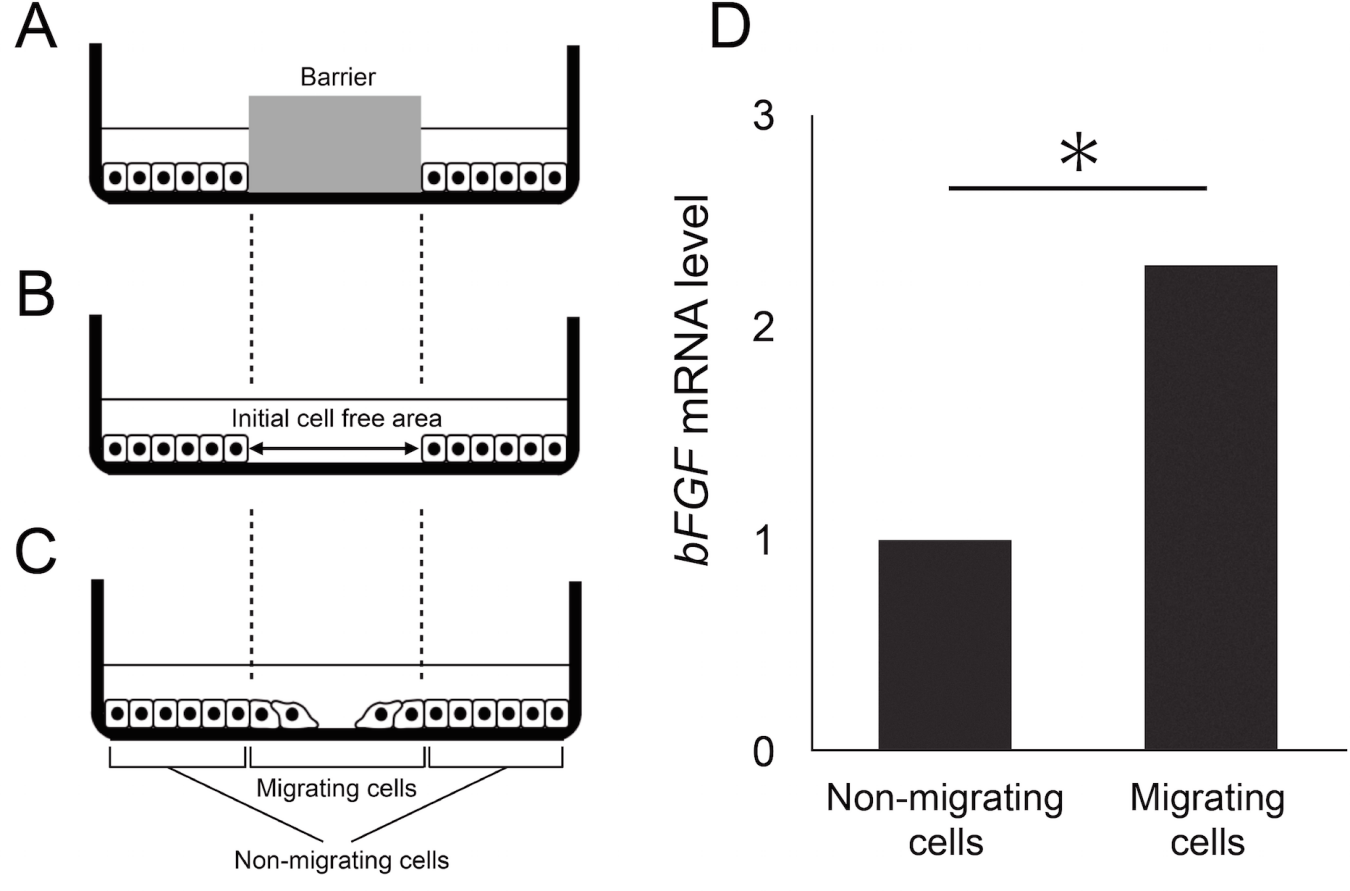
*bFGF* expression in migrating and non-migrating cells. A-C: Schematic images of cell migration assay. As shown in C, the cells within the initial cell-free area were defined as “migrating cells” and the cells outside the initial cell-free area were defined as “non-migrating cells”. D: mRNA expression in non-migrating cells and migrating cells. *P < 0.05.

**Fig 6:**
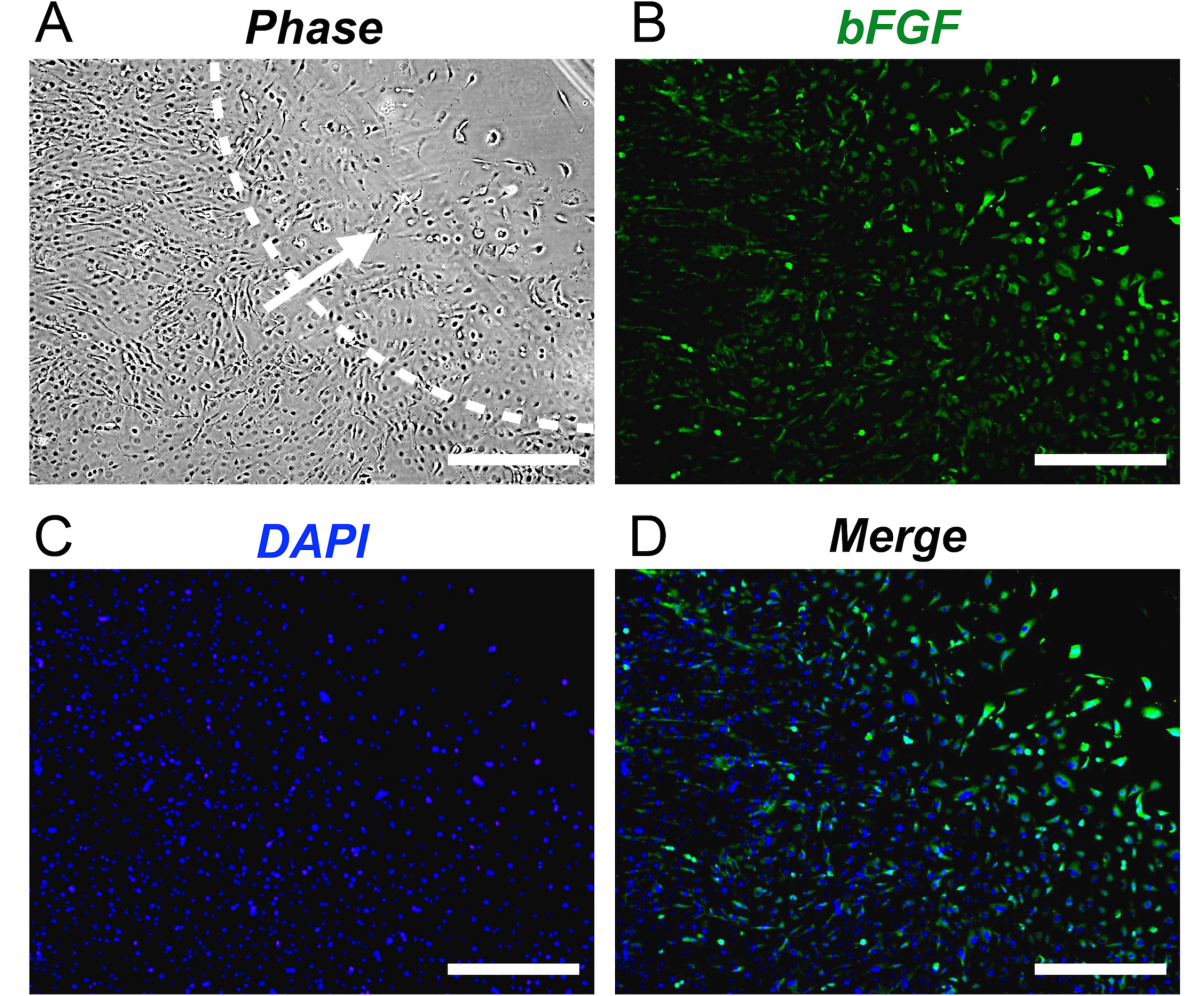
bFGF levels in migrating MIO-M1 cells. A: Phase contrast image of migrating MIO-M1 cells. The dotted line indicates the margin of the initial cell-free area. The arrow indicates the direction of cell migration. B: bFGF immunostaining (green) indicates that the cells at the leading edge of the migrating cells had a higher degree of staining than non-migrating cells. C: DAPI (blue) was used to show cell nuclei. D: Merged immunofluorescence micrograph of B and C. Bars = 500 μm.

Müller cells are known to produce bFGF when activated and subsequently proliferate and migrate [13]. Therefore, we investigated the effect that migration has on bFGF production in MIO-M1 cells using a cell exclusion zone assay. In order to harvest migrating and non-migrating cells separately, we used a larger sized culture dish and barrier (60-mm diameter and 15-mm diameter, respectively). We then removed the barrier to promote cell migration. When the cell-free area reached a diameter of approximately 7.5 mm, we were able to harvest both migrating and non-migrating cells separately and to measure *bFGF* mRNA levels using real time RT-PCR (Fig 5A, 5B and 5C). Briefly, before harvesting cells, we prepared a paper on which concentric circles with diameters of 60 mm and 15 mm were drawn. In order to distinguish between migrating and non-migrating cells, we placed the dish on the paper so that the dish overlapped with the 60-mm diameter circle. We then scraped the cells in the 15-mm circle with micropipette tips and immediately suctioned the detached cells and collected these as migrating cells. In the same way, we scraped the cells located outside the 15-mm diameter circle and collected these as non-migrating cells. As shown in Fig 5D, *bFGF* mRNA levels were significantly higher in migrating cells compared to non-migrating cells (*P* = 0.03). Next, in order to assess whether there were differences in bFGF protein levels, we performed immunostaining of the cells using a bFGF antibody. The results of this analysis indicate that the migrating cells had higher amounts of bFGF than the non-migrating cells (Fig 6B and 6D).

## Discussion

For many years, the only widely available method to study cell migration was a 2D cell migration assay known as the scratch wound assay. However, this assay destroys any ECM coating, and it thus cannot be used to effectively investigate the ECM and cell migration. In recent years, cell exclusion zone assays have been introduced as a method to overcome this drawback [12]. This commercially available assay excludes cells from attaching to a central zone through the use of silicone-based stoppers and a self-dissolving biocompatible gel [9,10,12]. The kits that are sold to perform this assay do not allow the use of a wide range of ECM coatings. In addition, the only available barrier is 2 mm in diameter and circular, and thus this assay can currently only be used to investigate migration area. Use of these kits are also limited because they are expensive [9]. Therefore, in order to investigate the effect that extracellular matrix proteins have on cell migration, we devised a cell exclusion zone assay that utilizes RTV silicone rubber as the barrier material. Our results indicate that the use of RTV silicone rubber provides multiple benefits. Firstly, this barrier allows for the investigation of the effects of any ECM component of interest on cell migration. Secondly, because we were able to increase the scale of the cell exclusion zone assay by making the barrier larger, we could perform both multi-parametric analysis, such as comparisons of gene expression (Fig 5), as well as immunostaining in migrating and non-migrating cells (Fig 6). Finally, our method allows cell migration assays to be performed at a lower cost. Therefore, our newly developed assay allows for low-cost investigation of several variables that cannot be investigated using the conventional assay.

We aimed to develop a method by which researchers can create a barrier at a low cost, and we sought to determine which material would be suitable for use as a barrier. Our results indicate that the RTV silicone rubber KE-3495-T is well suited as a barrier material. As mentioned above, conventional cell exclusion zone assays utilize silicone-based seeding stoppers, a self-dissolving biocompatible gel, or biocompatible plastic [9–12]. However, the manufacture of these barriers requires specialized machinery and molds, which makes them technically and financially difficult to manufacture in the laboratory. We sought a material with the following properties: 1) A high degree of biocompatibility, 2) A simple manufacturing process 3) Easily manipulated 4) Good adhesion to the culture dish but also easy to remove completely, and 5) Low-cost. RTV silicone rubber is a type of silicone that hardens as it absorbs moisture from the atmosphere. It is also highly biocompatible, does not produce harmful substances during the hardening process, and it can be sterilized in an autoclave. RTV silicone rubber can also be formed into a variety of sizes and shapes according to its intended use after hardening, and its price in Japan is approximately US$ 3.00 for an amount sufficient to manufacture 100 cylindrical barriers with diameters of 4 mm each. We investigated three types of RTV silicone rubber with differing viscosities and found that, in comparison to the others, KE-3495-T hardened the most uniformly. This property gave it an extremely smooth post-hardened surface, and it was thus highly adherent to the culture dish. KE-3495-T was also able to be completely removed from the culture dish, and it did not damage the ECM coating on the dish (Fig 3). Its highly adherent and easy to remove properties were very reproducible in actual migration assays, as can be seen in the small SE in Fig 4Q.

This assay has several limitations. Firstly, only adhesion cells, but not floating cells, can be analyzed. In addition to MIO-M1 cells, we confirmed that this assay could be used to analyze the migration of other cells such as vascular endothelial cell (S1 Fig). Secondly, this assay is not suitable for high-throughput tests. Finally, it remains unknown how long the barrier can be stored and still maintain its original adhesiveness, although we did confirm that the barrier could be used at least 1 month after its manufacture.

In summary, we developed a method to make a barrier using RTV silicone rubber and showed its ability to be effectively used in the cell exclusion zone assay. We were thus able to perform multi-parametric analysis of cell migration at a low cost.

## Supporting information

S1 Video The technique of a novel cell exclusion zone assay with a barrier made from room temperature vulcanizing silicone rubber. (MOV)

S1 Fig Cell exclusion zone assays with RTV silicone rubber for HUVEC. A-H: Phase contrast microscopic images of human umbilical vein endothelial cells (HUVEC) that have undergone a migrating assay in an uncoated cell culture dish. A-D show an image that was photographed immediately after removing the barrier, while E-H show an image taken 48 hours after removing the barrier. C and G each show an enlarged section of A and E, respectively. Each dotted line in B, D, F, and H indicates the margin of the cell-free area in the image to the left of each of these panels. Bars = 1 mm.
(TIF)
