# Supplementary figures and images for: A novel cell exclusion zone assay with a barrier made from room temperature vulcanizing silicone rubber
Source: PLoS One. 2017 Dec 21;12(12):e0190198. doi: 10.1371/journal.pone.0190198 (PMC5739469; doi:10.1371/journal.pone.0190198)

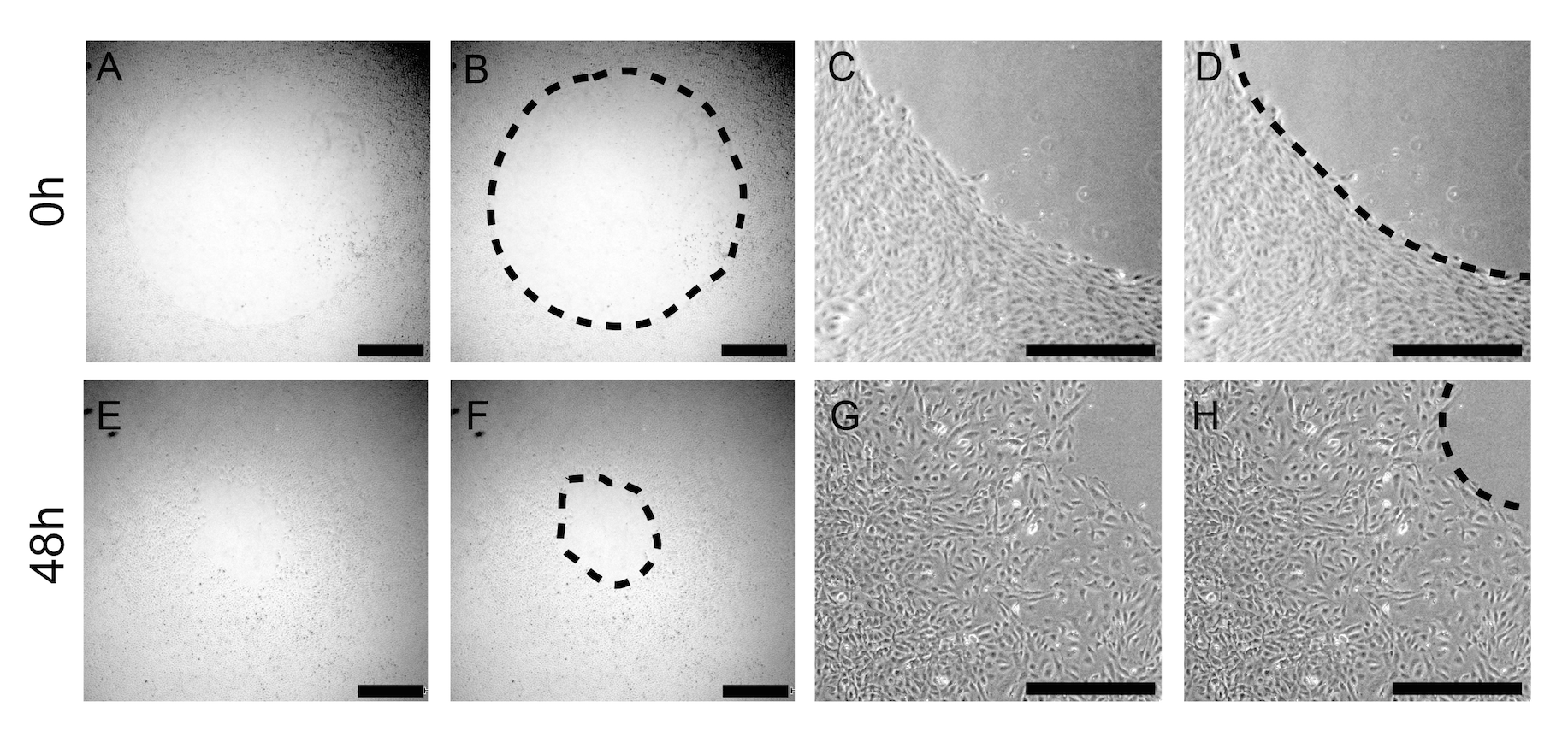

Supplement: S1 Fig — A-H: Phase contrast microscopic images of human umbilical vein endothelial cells (HUVEC) that have undergone a migrating assay in an uncoated cell culture dish. A-D show an image that was photographed immediately after removing the barrier, while E-H show an image taken 48 hours after removing the barrier. C and G each show an enlarged section of A and E, respectively. Each dotted line in B, D, F, and H indicates the margin of the cell-free area in the image to the left of each of these panels. Bars = 1 mm. (TIF) [file pone.0190198.s002.tif]
